# Absence of lenadogene nolparvovec DNA in a brain tumor biopsy from a patient in the REVERSE clinical study, a case report

**DOI:** 10.1186/s12883-022-02787-y

**Published:** 2022-07-12

**Authors:** Nancy J. Newman, Matthew Schniederjan, Pia R. Mendoza, David J. Calkins, Patrick Yu-Wai-Man, Valérie Biousse, Valerio Carelli, Magali Taiel, Francois Rugiero, Pramila Singh, Alexandra Rogue, José-Alain Sahel, Philippe Ancian

**Affiliations:** 1 Departments of Ophthalmology, Neurology and Neurological Surgery, Neuro-Ophthalmology Unit, Emory Eye Center, Emory University School of Medicine, 1365-B Clifton Road NE, Atlanta, GA 30322 USA; 2 Department of Pathology and Laboratory Medicine, Emory University, Atlanta, GA USA; 3 Department of Ophthalmology and Visual Sciences, Vanderbilt University Medical Center, 1161 21st Avenue South, Nashville, TN 37232 USA; 4 Cambridge Centre for Brain Repair and MRC Mitochondrial Biology Unit, Department of Clinical Neurosciences, University of Cambridge, Cambridge, UK; 5 Cambridge Eye Unit, Addenbrooke’s Hospital, Cambridge University Hospitals, Cambridge, UK; 6 Moorfields Eye Hospital, London, UK; 7 UCL Institute of Ophthalmology, University College London, London, UK; 8 IRCCS Istituto delle Scienze Neurologiche di Bologna, UOC Clinica Neurologica, Bologna, Italy; 9 Unit of Neurology, Department of Biomedical and Neuromotor Sciences, University of Bologna, Bologna, Italy; 10 GenSight Biologics, 74 rue du Faubourg Saint Antoine, 75012 Paris, France; 11 Diamond Pharma Services - ProPharma Group, Harlow, CM20 2FB UK; 12 Charles River Laboratories, Evreux, France; 13 Sorbonne Université, INSERM, CNRS, Institut de la Vision, Paris, France; 14 Fondation Ophtalmologique A. de Rothschild, Paris, France; 15 Department of Ophthalmology, The University of Pittsburgh School of Medicine, Pittsburgh, PA USA; 16 CHNO des Quinze-Vingts, Institut Hospitalo-Universitaire FOReSIGHT, INSERM-DGOS CIC, Paris, France

**Keywords:** Case report, Viral vector transduction, Recombinant adeno-associated virus vector 2 serotype 2, *ND4*, qPCR assay, Lenadogene nolparvovec, Leber hereditary optic neuropathy

## Abstract

**Background:**

Leber Hereditary Optic Neuropathy (LHON) is a rare, maternally-inherited mitochondrial disease that primarily affects retinal ganglion cells (RGCs) and their axons in the optic nerve, leading to irreversible, bilateral severe vision loss. Lenadogene nolparvovec gene therapy was developed as a treatment for patients with vision loss from LHON caused by the most prevalent m.11778G > A mitochondrial DNA point mutation in the *MT-ND4* gene. Lenadogene nolparvovec is a replication-defective recombinant adeno-associated virus vector 2 serotype 2 (AAV2/2), encoding the human wild-type MT-ND4 protein. Lenadogene nolparvovec was administered by intravitreal injection (IVT) in LHON patients harboring the m.11778G > A ND4 mutation in a clinical development program including one phase 1/2 study (REVEAL), three phase 3 pivotal studies (REVERSE, RESCUE, REFLECT), and one long-term follow-up study (RESTORE, the follow-up of REVERSE and RESCUE patients).

**Case presentation:**

A 67-year-old woman with *MT-ND4* LHON, included in the REVERSE clinical study, received a unilateral IVT of lenadogene nolparvovec in the right eye and a sham injection in the left eye in May 2016, 11.4 months and 8.8 months after vision loss in her right and left eyes, respectively. The patient had a normal brain magnetic resonance imaging with contrast at the time of diagnosis of LHON. Two years after treatment administration, BCVA had improved in both eyes. The product was well tolerated with mild and resolutive anterior chamber inflammation in the treated eye. In May 2019, the patient was diagnosed with a right temporal lobe glioblastoma, IDH-wildtype, World Health Organization grade 4, based on histological analysis of a tumor excision. The brain tumor was assessed for the presence of vector DNA by using a sensitive validated qPCR assay targeting the ND4 sequence of the vector.

**Conclusion:**

ND4 DNA was not detected (below 15.625 copies/μg of genomic DNA) in DNA extracted from the brain tumor, while a housekeeping gene DNA was detected at high levels. Taken together, this data shows the absence of detection of lenadogene nolparvovec in a brain tumor (glioblastoma) of a treated patient in the REVERSE clinical trial 3 years after gene therapy administration, supporting the long-term favorable safety of lenadogene nolparvovec.

## Background

Leber Hereditary Optic Neuropathy (LHON) is a rare, maternally-inherited mitochondrial disease that primarily affects retinal ganglion cells (RGCs) and their axons in the optic nerve, leading to irreversible, bilateral, and often sequential severe vision loss [1]. The m.11778G > A point mutation in the mitochondrial DNA (mtDNA) *MT-ND4* gene is the most prevalent variant causing the disease and is associated with a poor visual prognosis [2, 3].

Lenadogene nolparvovec gene therapy was developed as a treatment for LHON patients affected by the m.11778G > A *MT-ND4* mtDNA mutation. The *MT-ND4* gene is normally encoded by the mitochondrial genome and expressed and translated by the intramitochondrial machinery.

Lenadogene nolparvovec is a recombinant replication-defective single-stranded deoxyribonucleic acid (DNA) adeno-associated viral vector 2 serotype 2 (AAV2/2). It contains a codon-optimized complementary DNA (cDNA) that encodes the human wild-type mitochondrial ND4 protein under the control of a cytomegalovirus immediate early promoter in an intron-containing expression cassette (beta globin intron, HBB2) flanked by the viral inverted terminal repeats from the vector. The construct includes the cis-acting elements of human cytochrome c oxidase 10 (COX10) mitochondrial ribonucleic acid (mtRNA), thus ensuring efficient delivery of the corresponding hybrid mRNA to the mitochondrial surface where translation of the human ND4 protein occurs coupled with mitochondrial translocation [4, 5].

Hence, lenadogene nolparvovec promotes allotopic expression of the wild type *MT-ND4* gene in the RGC nucleus, leading to mRNA cytoplasmic translation of the MT-ND4 subunit, which is ultimately imported within mitochondria to assemble into Complex I, thereby rescuing respiratory chain function, as shown in cell and animal models [4, 6–10].

Lenadogene nolparvovec has been administered in m.11778G > A *ND4* LHON patients by intravitreal injection (IVT) in a clinical development program including one phase 1/2 study (REVEAL) [11] and three phase 3 pivotal studies (REVERSE, RESCUE, REFLECT) [12, 13]. In total, 189 *MT-ND4* patients were treated with a single unilateral or bilateral IVT of lenadogene nolparvovec with no concerning safety issues other than usually mild intraocular inflammation controlled with topical corticosteroids in most cases. Here, we report on the safety of lenadogene nolparvovec based on the analysis of an excised specimen of brain tumor from a LHON patient carrying the m.11778G > A mutation, who had been monitored for 3 years after unilateral injection with lenadogene nolparvovec in the REVERSE study.

## Case presentation

### Objective of the analysis of the tumor excision

The aim of the study was to assess lenadogene nolparvovec safety, by evaluating the possible presence of lenadogene nolparvovec viral genomes in a tumor excision and if found, to confirm the absence of integration of lenadogene nolparvovec DNA into tumor cells. Informed consent was given by the patient for use of the data related to the excision for scientific research.

### Patient history

A 67-year-old woman with *MT-ND4* LHON, included in the REVERSE clinical study, received a unilateral IVT of lenadogene nolparvovec in the right eye and a sham injection in the left eye in May 2016, 11.4 months and 8.8 months after vision loss in her right and left eyes, respectively. The patient had a normal brain magnetic resonance imaging with contrast at the time of diagnosis of LHON. Before injection, the patient’s best-corrected visual acuity (BCVA) was off-chart, assessed as “counting fingers” in both eyes. 2 years after treatment administration, BCVA had improved in both eyes, with a BCVA of 20/160 and 20/400 for the lenadogene nolparvovec- and sham-treated eyes, respectively. The improvement from nadir was - 1.3 LogMAR for the treated eye, and - 0.9 LogMAR for the sham eye. The product was well tolerated with mild and resolutive anterior chamber inflammation in the treated eye. After completing the two-year REVERSE study, the patient enrolled in the long-term RESTORE study for further follow-up. In May 2019, 3 years after unilateral treatment administration, the patient was diagnosed with a right temporal lobe glioblastoma, IDH-wildtype, World Health Organization grade 4, based on histological analysis of a tumor excision which showed a high-grade infiltrating astrocytoma with necrosis and microvascular proliferation. The brain tumor was assessed and considered to be unrelated to the study drug or the study procedure.

### Samples and sample processing

The tumor excision was performed on the patient and the tissue was then fixed for 22 hours in 10% neutral buffered formalin, paraffin embedded and stored at room temperature until dispatched to Charles River Laboratories (Evreux, France) for analysis and quantitation of any lenadogene nolparvovec DNA present in the sample. Three tumor excision blocks were analyzed.

DNA was extracted from each tumor excision block in triplicate (3 × 10 μm slices) using the QIAamp® DNA FFPE Tissue Kit (Qiagen, UK) and then pooled to obtain 3 pooled samples. After extraction, DNA concentration and purity (OD ratios) were determined by UV spectrophotometry using a Nanodrop 8000 instrument (Thermo Fischer Scientific, UK). DNA concentration was determined using a Qubit Flex Fluorometer (Thermo Fischer Scientific, UK) and DNA distribution was checked by micro capillary electrophoresis using the Agilent DNA 1000 kit (Agilent, USA).

### Feasibility of DNA extraction from formalin-fixed paraffin-embedded excision blocks

To confirm that DNA of adequate quality could be extracted from formalin-fixed paraffin-embedded (FFPE) samples, DNA was extracted from 5 *cynomolgus* monkey brain samples (i.e. including brain cortex or pons/cerebellar) embedded in paraffin under similar conditions as the patient’s tumor excision samples. All 5 monkey samples had DNA concentrations (cynomolgus monkey *ACTB* gene) between 44.65 and 235.5 ng/μL. The PCR (polymerase chain reaction) assay for the amplification of the housekeeping gene produced Ct (threshold cycle) values between 25.92 and 29.93, showing that the DNA was of adequate quality for PCR amplification.

DNA was extracted from a positive control sample (i.e. rat liver) to monitor the DNA extraction process. DNA was successfully extracted from the positive control showing that no issues occurred during the extraction process.

DNA was then extracted from the patient tumor excision. Following extraction, triplicate samples were pooled and DNA concentration was measured. *Homo sapiens actin beta* (*ACTB*) was used as the housekeeping gene to demonstrate that the quality of DNA extracted from FFPE blocks was adequate for PCR amplification.

### qPCR analysis

The detection of lenadogene nolparvovec DNA was evaluated by qPCR (quantitative polymerase chain reaction) using primers specific for the human *ND4* sequence. Specificity of the assay and matrix effects were checked using DNA extracted from human blood samples taken from six individuals and spiked with pAAV2/2-ND4. Viral genomes were analyzed using a validated qPCR assay targeting the *ND4* transcript region. The following assay validation parameters were determined: dynamic range, detection limit, quantification limits, within-run and between-run precision and accuracy, dilution integrity, matrix effect, selectivity and specificity in human genomic DNA. The DNA extraction recovery yield from human blood after spiking with known amounts of lenadogene nolparvovec was determined and the stability of lenadogene nolparvovec DNA in these samples after storage at − 20 °C was evaluated. Table 1 shows the qPCR assay validation parameters.

**Table 1:** qPCR assay validation parameters

Matrix Calibration curve and regression	Herring sperm DNA, 400 ng per PCR well Pass - Calibration standards ranging from 100 to 1 × 107 pAAV2/2-ND4 copies/well, fitted with a linear regression			
Within-run precision and accuracy	Pass - CV% < 15.0, absolute RE% < 8.0			
	QC level	Nominal concentration (copies/well)	CV%	RE%
	QC ULOQ	1.00E+ 07	11.1	−0.1
	QC high	7.50E+ 06	8.0	−0.8
	QC mid	1.05E+ 04	6.2	−3.5
	QC low	3.00E+ 02	6.6	−7.2
	QC LLOQ	1.00E+ 02	14.4	−2.0
Between run precision and accuracy	Pass - CV% < 9.0, absolute RE% < 6.0			
	QC level	Nominal concentration (copies/well)	CV%	RE%
	QC ULOQ	1.00E+ 07	6.9	−3.1
	QC high	7.50E+ 06	4.4	0.1
	QC mid	1.05E+ 04	4.9	5.4
	QC low	3.00E+ 02	8.2	4.2
	QC LLOQ	1.00E+ 02	4.1	2.7
Dynamic range of the assay	100 to 1 × 10^7^ copies/well corresponding to 250 to 2.5 × 10^7^ copies/μg of DNA			
Detection limit of the assay	6.25 copies/well corresponding to 15.625 copies/μg of DNA			
Specificity of the assay	Pass - Tested with DNA extracted from blood samples from six humans			
Matrix effect	Pass - Tested with DNA extracted from the six human blood samples spiked with 10^4^ copies of pAAV2/2-ND4			

*pAAV2/2-ND4* Plasmid containing the sequence for AAV2/2-ND4 DNA, *CV%* Percentage of Coefficient of variation, *RE%* Percentage of Relative Error, *ULOQ* Upper Limit of Quantification, *LLOQ* Lower Limit of Quantification.

In parallel, a DNA quality control was performed by assessing gene levels of a housekeeping gene, human actin beta (*ACTB*; Hs03023880_g1, Thermofisher), to check that the quality of the extracted DNA was adequate for PCR amplification and to ensure that any negative results for the *ND4* sequence were true negatives. PCR was performed on 96-well plates, using a specific set of primers and probe:

- forward primer: ND4-F 5′- TCCTGAAGCTGGGTGGTTATG − 3′,
- reverse primer: ND4-R 5′- GGCTCTTGAGGTCAGTCTGCC − 3′,
- internal Taqman® probe: ND4b1_MGB.P FAM 5′- CATGGCTTACCCTTTC -3′ MGB.

All samples were spiked with 2.5 × 103 copies of exogenous DNA (*Drosophila melanogaster* Antp gene), and then assayed by qPCR. The recovery percentage of the measured Antp copy number had to be lower than 50% of the nominal value to consider the PCR as inhibited [14].

### Feasibility of DNA extraction from formalin-fixed paraffin-embedded excision blocks

All pooled samples had DNA concentrations between 31.00 and 69.60 ng/μL. Therefore, the impact of any potential contamination was considered to be negligible as none of the samples had PCR inhibition.

### Test compound detection by qPCR analysis

Lenadogene nolparvovec was not detected [below the limit of detection (BLD)] in any samples using the *ND4* qPCR assay. No PCR inhibition was detected in any sample. All sentinel controls had BLD values, showing that no cross-contamination between samples occurred during the extraction or qPCR procedures.

## Discussion

This analysis showed that a sufficient quantity of DNA was extracted from tumor excision blocks embedded in paraffin originating from an *MT-ND4* LHON patient. DNA from a housekeeping gene was amplified successfully by PCR from tumor slides, indicating that the DNA quality was adequate for PCR amplification. Lenadogene nolparvovec DNA was not detected in glioblastoma tumor samples from the patient 3 years post-administration of lenadogene nolparvovec, and no PCR inhibition was detected. These results, along with the data accumulated in the five clinical trials conducted with lenadogene nolparvovec to date, demonstrate the long-term safety of lenadogene nolparvovec gene therapy as regards the potential for causing tumoral transformation.

Data from a study in *cynomolgus* monkeys have shown that lenadogene nolparvovec DNA penetrates the central nervous system (CNS) visual pathways (optic chiasm, optic tract and contralateral optic nerve) following intravitreal administration [12, 14]. As lenadogene nolparvovec is a non-integrating, replication defective vector, vector DNA detected in CNS structures originates from episomal vector DNA and thus, when the passage of vector DNA into the brain occurs, it does not result in vector integration into the genome where further interaction with oncogenes might occur. With regards to immune reactions to the capsid, there have been no reports of long-term expression of intact rAAV particles in the CNS following intraocular administration of rAAV-based gene therapy; only long-term expression of rAAV-delivered transgenes has been reported. The available evidence indicates that intravitreal injection is more likely to distribute rAAV vectors to the CNS than subretinal injection [15, 16]. Following IVT of rAAV2/2 in dogs, rAAV sequences were detected along the visual pathways: optic nerve, optic chiasm, optic tract, lateral geniculate nucleus, colliculus, optical radiation and visual cortex [15]. These data suggest that IVT can lead to anterograde vector genome distribution along the axons of the ganglion cells to the brain. However, so far, there have been no reports of CNS toxicity in preclinical or clinical studies using intravitreal delivery of rAAV vectors, even after long periods of viral vector expression. Indeed, the duration of AAV-delivered transgene expression following just a single dose is essentially permanent in nondividing cells. AAV-delivered transgenes are expressed for more than 6 months in the mouse brain [17] and can persist in other tissues for at least 6 years in primates [18] and at least 8 years in dogs [19, 20]. Importantly, a gene therapy trial has shown that the therapeutic effects of AAV-delivered transgenes can persist for at least 10 years in the human brain [21], and post-mortem analyses in human brain 10 and 8 years post rAAV2 administration directly into the brain (at doses of 1.8 × 10^11^ vg and 2.4 × 10^12^ vg, respectively) have demonstrated a lack of CNS toxicity despite long term expression of AAV genome [22].

These data agree with other results accumulated in several intraocular gene therapy programs currently in progress. For example, the University of Miami and the National Eye Institute in the US are developing an rAAV2/ND4 therapy for the treatment of LHON (ScAAV2-P1ND4v2). The 3-year interim results from 14 patients who received a low dose of 1.18 × 10^9^ vg and a medium dose of 5.81 × 10^9^ vg reported that there were no CNS concerns [23]. Similarly, the Huazhong University of Science and Technology in Wuhan, China, has also developed an rAAV2/ND4 vector for IVT in LHON patients with the LHON m11778G > A mitochondrial DNA mutation. After 7 years of follow-up, no adverse events were reported among the 8 of 9 treated patients who completed the 75- to- 90-month follow-up visits. There were no systemic, CNS-related or ocular adverse events over this long term-follow-up period [24, 25].

The outcome of AAV-mediated retinal gene therapy greatly varies among the reported experimental, pre-clinical and clinical studies. While some demonstrate stable transgene expression for up to 6 years and no vector-related relevant immunological adverse events, others observe the induction of innate and adaptive immune responses and/or ocular inflammation that can be paralleled by a loss of transgene expression [26]. However, CNS toxicity has thus far never been reported.

AAV vectors exhibit an improved safety profile compared to other viral vectors because of their low integration frequency, low immunogenicity and the non-pathogenicity of their parental form. Accordingly, a number of rAAV vectors are being developed as delivery cargos for accessing the CNS [27, 28].

In the present investigation, no lenadogene nolparvovec DNA was detected in CNS tumor samples from a patient who developed a glioblastoma tumor 3 years after intravitreal administration of lenadogene nolparvovec in one eye. Although this does not demonstrate an absence of vector distribution to the CNS, it is supportive of the long-term safety of lenadogene nolparvovec. These results are consistent with the previously reported favorable safety profile of lenadogene nolparvovec and the absence of CNS adverse reactions [11–13].

## Conclusion

In total, 189 *MT-ND4* patients were treated with a single unilateral or bilateral IVT of lenadogene nolparvovec with no safety issues or concerns in five clinical studies [11–13]. Here, we report the absence of lenadogene nolparvovec in human brain tumor excision tissue obtained 3 years after unilateral intravitreal treatment administration, when using a qPCR assay detection method specific for the human *ND4* gene. Thus, this study indicates that the tumor occurrence was unrelated to lenadogene nolparvovec. These results confirm the favorable safety profile of lenadogene nolparvovec gene therapy in *MT-ND4* LHON treated patients [11–13].

## Data Availability

The datasets used and/or analyzed during the current study are available from the corresponding author on reasonable request.

## Abbreviations

LHON: Leber Hereditary Optic Neuropathy
RGC: Retinal ganglion cells
mRNA: Messenger ribonucleic acid
mtDNA: Mitochondrial deoxyribonucleic acid
mtRNA: Mitochondrial ribonucleic acid
AAV2/2: Adeno-associated virus vector 2 serotype 2
IVT: Intravitreal injection
DNA: Deoxyribonucleic acid
cDNA: Codon-optimized complementary DNA
BCVA: Best-corrected visual acuity
PCR: Polymerase chain reaction
Ct: Threshold cycle
qPCR: Quantitative polymerase chain reaction
CNS: Central nervous system
rAAV: Recombinant adeno-associated virus
